# Brush-like Polyaniline with Optical and Electroactive Properties at Neutral pH and High Temperature

**DOI:** 10.3390/ijms23158085

**Published:** 2022-07-22

**Authors:** Alain Salvador Conejo-Dávila, Carlos Rafael Casas-Soto, Eider Pedro Aparicio-Martínez, David Chávez-Flores, Víctor Hugo Ramos-Sánchez, Rocio Berenice Dominguez, Velia Carolina Osuna, Anayansi Estrada-Monje, Alejandro Vega-Rios, Erasto Armando Zaragoza-Contreras

**Affiliations:** 1 Department of Engineering and Materials Chemistry, Centro de Investigación en Materiales Avanzados, S.C., Miguel de Cervantes No. 120, Complejo Industrial Chihuahua, Chihuahua 31136, Mexico; alain.conejo@cimav.edu.mx (A.S.C.-D.); carlos.casas@cimav.edu.mx (C.R.C.-S.); eider.aparicio@cimav.edu.mx (E.P.A.-M.); 2 Facultad de Ciencias Químicas, Universidad Autónoma de Chihuahua, Chihuahua 31125, Mexico; dchavezf@uach.mx (D.C.-F.); vramos@uach.mx (V.H.R.-S.); 3 CONACYT-CIMAV S.C., Miguel de Cervantes 120, Complejo Industrial Chihuahua, Chihuahua 31136, Mexico; berenice.dominguez@cimav.edu.mx (R.B.D.); velia.osuna@cimav.edu.mx (V.C.O.); 4 Centro de Innovación Aplicada en Tecnologías Competitivas, A.C. Calle Omega No. 201, Industrial Delta, León 37545, Mexico; aestrada@ciatec.mx

**Keywords:** poly(*N*-vinylcarbazole), RAFT-polymerization, brush-like polyaniline, neutral pH

## Abstract

In this research, a brush-like polyaniline (poly(2-acrylamide-2-methyl-1-propanesulfonate)-*g*-polyaniline)-*b*-poly(*N*-vinylcarbazole) (BL PAni) was developed as a strategy to overcome the limited processability and dedoping above pH 4 of conventional polyaniline (PAni). For the BL PAni synthesis, RAFT polymerization (homopolymer), RAFT-mediated surfactant-free emulsion polymerization (block copolymer), and interfacial oxidative polymerization were applied to graft the PAni chains. NMR and FT-IR spectroscopies were performed to confirm the structural elucidation of the reaction pathways, while the thermal properties were analyzed by TGA and DSC. Notably, the BL PAni presents absorption throughout the visible region and up to the near-infrared, showing dedoping resistance at up to 80 °C and at a neutral pH. The absorption range of the BL PAni, block copolymer, and homopolymer were studied by UV–Vis spectroscopy in solid-state and dispersion/solution, highlighting BL PAni and poly(anilinium 2-acrylamide-2-methyl-1-propanesulfonate)-*b*-poly(*N*-vinylcarbazole) (PAAMP-*b*-PVK) due to the π-stacking between the anilinium and carbazole groups. The cyclic voltammetry confirmed the persistence of electroactivity at a pH near 7.

## 1. Introduction

Polyaniline (PAni), particularly in its emeraldine salt form, presents characteristics such as conductivity, pseudocapacitance, synthetic versatility, accessibility to starting materials, and chemical stability, which are attractive for the development of devices with electrochemical applications [1]. However, some drawbacks have not been solved, such as low processability, insolubility, thermal dedoping, and dedoping at pH higher than 4 [2]. Several strategies have been used to counteract such disadvantages, e.g., composites [3], polymer blending [4], and others [5,6]. Monomers analogous to aniline are commonly utilized; for instance, the *N*-substituted anilines, e.g., *N*-methylaniline [7], or heterocycle-substituted anilines, e.g., *N*-vinylcarbazole [8].

In particular, poly(*N*-vinylcarbazole) is a thermoplastic optoelectronic polymer with photoconductivity [9], photoexcitation [10], and charge transfer properties (by electron [11] or hole [12] transference). In addition, PVK has been employed to develop OLED inks [13], photoconductive films [14], solar cells [15], and diodes [16]. The formation of π-stacking interactions due to the aromatic rings of PVK is responsible for these properties [17]. Furthermore, the PVK aromatic rings act as electron donors [10]. When this polymer is doped with electroattractor groups such as cation metals or aromatic compounds substituted with electroattractor functional groups such as NO_2_, F, COOR, and ^+^NR_3_, the face-to-face stack conformation is favored, which modifies the PVK optoelectronic properties [18,19,20].

Additionally, PAni/PVK composites have been widely studied for their simplicity and versatility; however, one disadvantage is that the PVK content in a typical formulation is high [21]. Basavaraja et al. demonstrated that PAni/PVK composites have semiconductor behavior owing to an increased conductivity while increasing the temperature compared with pure PVK [22], and Cadenas et al. reported a composite PMMA/PVK/PAni with a dedoping point close to pH 9 [21]. Furthermore, PVK improves the electroluminescence efficiency of quantum dot light-emitting diodes because PAni-PSS decreases PVK electronic energy levels [8,23].

Few publications on PAni-*co*-PVK random copolymers have been published [24]. For instance, Pratap et al. suggested using a PAni-*co*-PVK copolymer to elaborate electrochemical sensors because the copolymer presents a lower bandgap than homopolymers, according to their theoretical calculations [25].

Recently, the synthesis of polymeric architectures, especially graft copolymers [6,26,27], is a pathway that allows the modification or regulation of the properties of the conducting polymers [28]. For example, Massoumi et al. reported the synthesis of electroactive bottlebrush nano-copolymers of poly(3-(2-hydroxyethyl) thiophene)-*g*-PAni, which present a higher conductivity and thermal stability than poly(3-(2-hydroxyethyl)thiophene) [6]. In addition, PAni-*g*-alkyd resin (acrylic acid, methyl methacrylate, butyl acrylate, and glycidyl methacrylate) emulsions have demonstrated improved anti-corrosion properties compared to alkyd resin, PANI, and a PAni/alkyd resin blend [29]. Furthermore, the use of PAni graft copolymers in areas including corrosion [29], solar cells [30], and sensors [31] has been explored. The focus of these systems has been to improve conductivity, colloidal stability, and thermal stability; nevertheless, doping point and its applications around a neutral pH are limited.

The key research of this study was to develop a PAni with intrinsic properties such as an electroactive and doping point at neutral pH, absorption in the region visible, and optical stability at 80 °C in a brush block copolymer architecture. The PVK and poly(2-acrylamide-2-methyl-1-propanesulfonate) are constituents of the block copolymer. 

## 2. Results and Discussion

**Figure 1:**
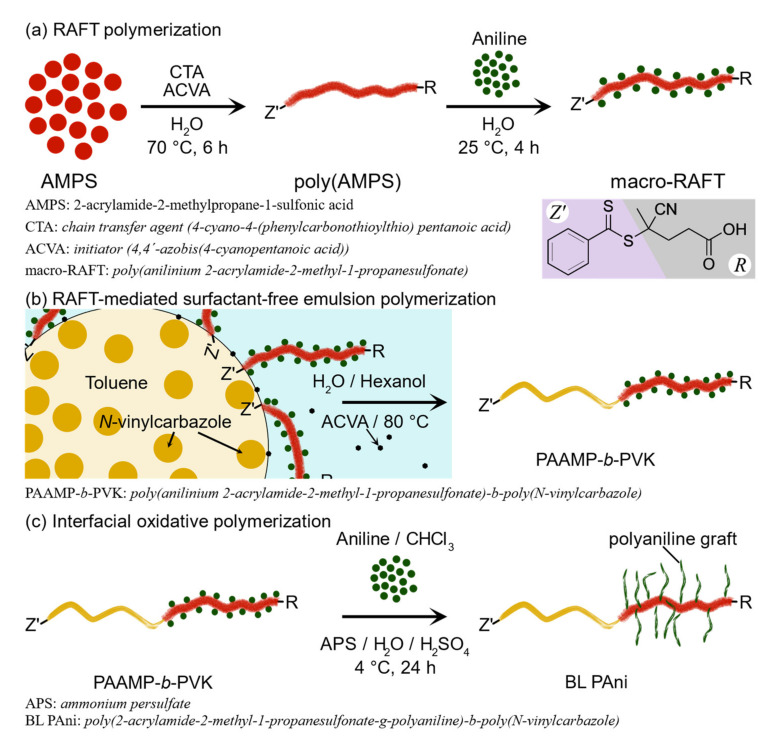
Sequence of polymerizations for the synthesis of brush-like polyaniline (BL PAni). (**a**) RAFT polymerization; (**b**) RAFT-mediated surfactant-free emulsion polymerization; (**c**) Interfacial oxidative polymerization.

A sequence of polymerizations was employed to synthesize brush-like polyaniline (BL PAni), as seen in Figure 1. The methodology followed to develop BL PAni commences with the RAFT polymerization of the monomer 2-acrylamide-2-methylpropane-1-sulfonic acid (AMPS), using the 4-cyano-4-(phenylcarbonothioylthio)pentanoic acid as the chain transfer agent (CTA) and 4,4′-azobis(4-cyanopentanoic acid) (ACVA) as the initiator in a relationship of [50:1:0.25]. Molar ratios were established from an analog polymer [32]. The next step is the generation of the graft point on the poly(2-acrylamide-2-methylpropane-1-sulfonic acid) poly(AMPS). Aniline reacts with the sulfonic acid contained in the polymer structure, forming poly(anilinium 2-acrylamide-2-methyl-1-propanesulfonate) (macro-RAFT). Figure 1a illustrates the RAFT polymerization scheme of the AMPS.

The poly(anilinium 2-acrylamide-2-methyl-1-propanesulfonate)-*b*-poly(*N*-vinylcarbazole) (PAAMP-*b*-PVK) copolymer was obtained by RAFT-mediated surfactant-free emulsion polymerization (Figure 1b). The fundamental principles of this technique are discussed by Zhou et al. [33]. The emulsion system is stabilized by macro-RAFT and a co-nonsolvent (hexanol) [34,35]. The co-nonsolvent aims to improve the interaction between the macro-RAFT and *N*-vinylcarbazole (NVK), allowing monomer propagation within the macro-RAFT, producing a hydrophobic segment. The copolymerization yield was determined by gravimetry, previously removing macro-RAFT, poly(*N*-vinylcarbazole) (PVK), and NVK with acetone. Table 1 provides the design of the PAAMP-*b*-PVK experiments, performing three formulations with different amounts of NVK and equal quantities of macro-CTAs.

The BL PAni system (Figure 1c) was developed using the amphiphilic block copolymer PAAMP-*b*-PVK(C) to facilitate its study and comparison with other systems. The anilinium ions contained along the PAAMP-*b*-PVK(C) hydrophilic segment were used as graft points for polyaniline (PAni) chains through an interfacial oxidative polymerization [36]. The PAni doping, according to the polymerization scheme shown in Figure 1c, is sulfuric acid.

### 2.1. Structural Characterization of the Polymerization Sequence

#### 2.1.1. Macro-RAFT

**Figure 2:**
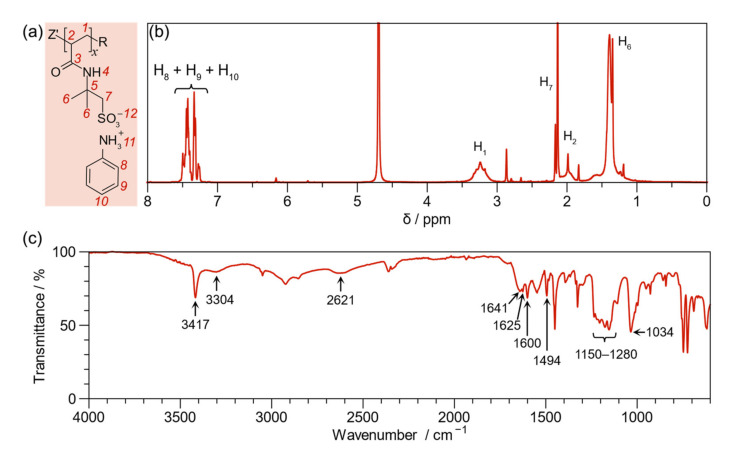
(**a**) Proton assignment of the poly(anilinium 2-acrylamide-2-methyl-1-propanesulfonate) (macro-RAFT) structure (**b**) ^1^H NMR spectrum of macro-RAFT; (**c**) FT-IR of macro-RAFT.

The macro-RAFT structural elucidation was performed by ^1^H NMR, ^13^C NMR, and FT-IR spectroscopy. The ^1^H NMR spectrum, as shown in Figure 2a, displays the aromatic proton signals of the anilinium ion located between 7.2 and 7.7 ppm (H_8_ + H_9_ + H_10_, 5H). In addition, the aliphatic proton peaks of the polymer backbone appear at 1.91 (H_2_, 1H) and 3.21 (H_1_, 2H) ppm. Likewise, the aliphatic protons corresponding to α-sulfonated methylene appear at 2.1 ppm (H_7_, 2H). Another signal corresponding to the methylene groups is at 1.4 ppm (H_6_, 6H). The pattern, position, and integration of signals are consistent with research previously reported by our group [37], except for the integration of protons corresponding to aromatic anilinium due to differences in synthesis methodology. In the previous work, the polymerization proceeded directly from a bifunctional monomer, including anilinium ions, while in the present research, the doping was performed post-polymerization. Figure S1 displays the ^1^H NMR (S1A), ^13^C NMR (S1B), and FT-IR (S1C) spectra of the macro-RAFT.

The macro-RAFT molecular weight (Mn) was determined from the ^1^H NMR spectrum [38]. The integration at 3.21 ppm and 2.64 ppm assigned to the methylene proton of the main chain and the methylene of 4-cyanopentanoic acid (CTA), respectively, were used. The degree of polymerization (DP) was 89, so the Mn was 18,355 g mol^−1^. On the other hand, the percentage of the graft points was 91%, determined by the relationship between the integration of the aromatic protons (H_8_, H_9_, H_10_) and the integration of α-sulfonated methylene (H_7_). Therefore, the number of graft points is 81, and the Mn of macro-RAFT is 25,981 g mol^−1^.

The ^13^C NMR spectrum (Figure S1B) displays the amide carbon peak at 212.54 ppm. All the aromatic carbon (anilinium) signals are also located between 130 and 120 ppm. The six aliphatic carbon peaks are below 65 ppm, at a high field.

The neutralization reaction, the graft point, was extensively analyzed employing FT-IR spectroscopy (Figure 2b). The molecular vibrations associated with anilinium and sulfonate anions are responsible for this bond; specifically, the stretching vibrations of the cationic amine group (^+^N-H) and sulfonate group (S=O) appear at 3304 cm^−1^ and 1034 cm^−1^ (symmetric SO_3_), respectively. Other peaks of cationic amine groups appear at 2621 cm^−1^ (overtone) and 1625 cm^−1^ (asymmetric NH_3_^+^ deformation vibration). It is important to note an overlap of the NH_3_^+^ rocking vibration and the asymmetric SO_3_ vibration in the region from 1150 cm^−1^ to 1280 cm^−1^. The bands corresponding to the anilinium aromatic ring (C=C) appear at 1600 and 1494 cm^−1^. The spectrum presents the stretching vibrations of N-H (3417 cm^−1^) and C=O (1641 cm^−1^) bonds, corresponding to the amide functional group [39]. These results confirm the coordinated covalent bond formation and, thus, the graft point. Figure S1C presents the FT-IR spectrum of macro-RAFT, pointing out the mentioned bands.

#### 2.1.2. Block Copolymer

**Figure 3:**
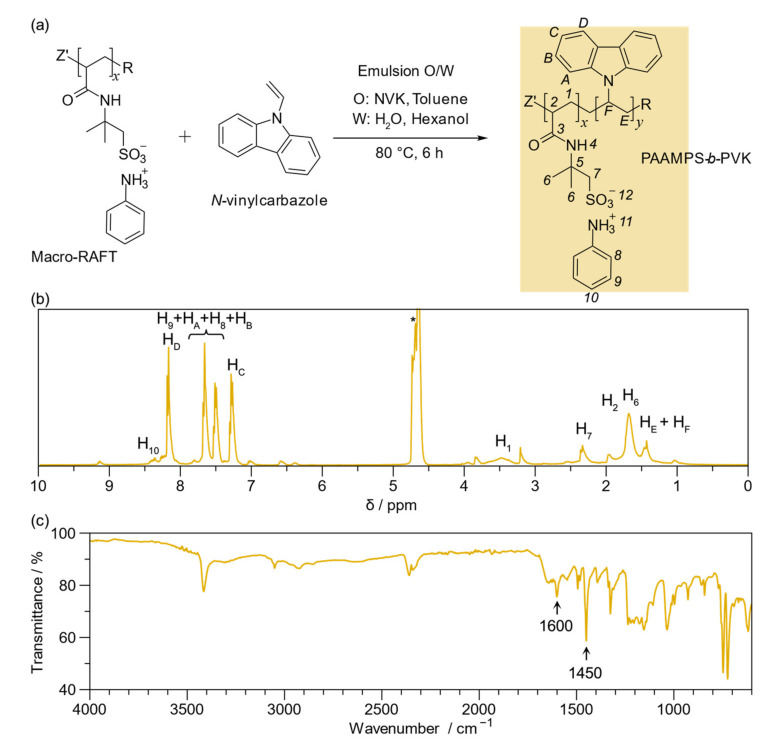
(**a**) RAFT-mediated surfactant-free emulsion polymerization scheme of *N*-vinylcarbazole (NVK) and proton assignment (yellow shaded area); (**b**) ^1^H NMR spectrum of poly(anilinium 2-acrylamide-2-methyl-1-propanesulfonate)-*block*-Poly(*N*-vinylcarbazole) PAAMP-*b*-PVK [1.0:1.5], * solvent: THF/water mixture; (**c**) FT-IR spectrum of PAAMP-*b*-PVK [1.0:1.5].

The PAAMP-*b*-PVK was analyzed in the same way as the macro-RAFT. Figure 3a illustrates the polymerization scheme of PAAMP-*b*-PVK via RAFT-mediated surfactant-free emulsion polymerization. Figure 3b shows the ^1^H NMR spectrum of the block copolymer. As seen, a doublet at 8.15 ppm (2H), another doublet at 7.65 ppm (2H), a triplet at 7.50 ppm (2H), and a triplet at 7.27 ppm (2H) are present. This peak pattern is typical of the carbazole heterocycle [40] and partially overlaps with the anilinium signals already described. PVK aliphatic protons appear in the region from 1.0 to 2.0 ppm. The spectra also present all the proton signals of macro-RAFT already described. Figure S2A shows the integrated and assigned ^1^H NMR spectrum of PAAMP-*b*-PVK(C).

Moreover, the Mn of the three synthesized block copolymers was determined by ^1^H NMR spectroscopy. The spectra are altogether different, specifically in the H_C_ proton (carbazole group) of the PVK block. A significant correlation exists in each experiment between the integration of H_1_ (2H) attributed to the macro-RAFT segment and the integration of H_C_ (1H). The H_C_ proton does not overlap with the anilinium ion peaks. The macro-RAFT block is constant in all PAAMP-*b*-PVK copolymers, i.e., 89 DP, so the DP of the PVK block can be established. Hence, the Mn of the three formulations was calculated (Table 2).

Table 3 illustrates a comparison of the theoretical Mn and that calculated by NMR. The different relationships of PAAMP-*b*-PVK(A), PAAMP-*b*-PVK(B), and PAAMP-*b*-PVK(C) allowed us to visualize which one presents an acceptable behavior. However, for the purpose of this research, it is not necessary to have control over the polydispersity index. The appropriate molar ratio was 41.5 NVK to 1 macro-RAFT according to PAAMP-*b*-PVK(B).

The ^13^C NMR spectrum (Figure S2B) presents the six characteristic peaks corresponding to carbazole heterocycle substituted at 110, 118, 119, 122, 125, and 125 ppm. In addition, the spectra present the aliphatic carbon signals attributed to the polymer backbone at 45.01 and 42.13 ppm [41]. These signals correspond to the PVK block. The spectrum also presents the peaks of the macro-RAFT segments.

The FT-IR spectrum of the block copolymer, seen in Figure 3c, presents the peaks previously described for the macro-RAFT segment and the bands assigned to the symmetric and asymmetric stretching of C=C carbazole rings at 1600 and 1450 cm^−1^, respectively [42]. Figure S2C presents the FT-IR spectrum of PAAMP-*b*-PVK, pointing out the mentioned bands. Figures S3 and S4 illustrate the ^1^H NMR, ^13^C NMR, and FT-IR spectra of PAAMP-*b*-PVK(A) and PAAMP-*b*-PVK(C), respectively.

#### 2.1.3. Poly(2-acrylamide-2-methyl-1-propanesulfonate-graft-polyaniline)-Block-Poly(*N*-vinylcarbazole), BL PAni

**Figure 4:**
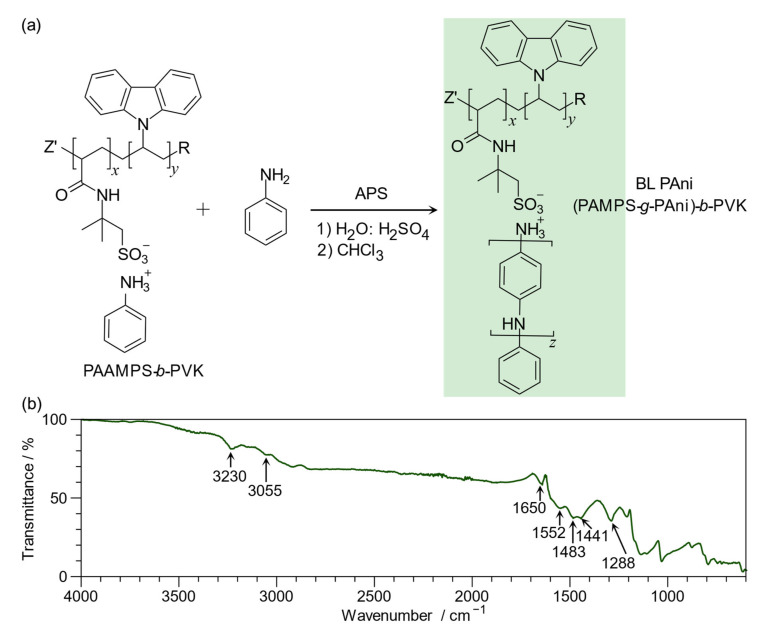
(**a**) Interfacial oxidative polymerization of aniline (Ani); (**b**) FT-IR spectrum of poly(2-acrylamide-2-methyl-1-propanesulfonate-*graft*-polyaniline)-*block*-poly(*N*-vinylcarbazole) (BL PAni).

The PAAMP-*b*-PVK(C) is slightly soluble in water and alcohol, facilitating its application as an ink. Nonetheless, NMR characterization was not possible, only FT-IR. Figure 4b shows the FT-IR spectrum of BL PAni. The characteristic peaks of PAni corresponding to stretching vibrations of the quinoid ring at 1582 cm^−1^ and the benzenoid ring at 1483 cm^−1^ were observed. It is worth noting that PAni exhibits the emeraldine oxidation state because the intensity is higher in the benzenoid ring than in the quinoid ring [43]. The spectrum also shows a band at 1288 cm^−1^ assigned to the bond C-N formed through oxidative polymerization [44].

In contrast, the PVK segment presents a molecular vibration at 1441 cm^−1^ and 3055 cm^−1^ assigned to the stretching C-H and C=C of the carbazole aromatic ring. In addition, the carbonyl stretching vibration of the amide group (II) appears at 1650 cm^−1^, and the N-H stretching vibration at 3230 cm^−1^. Likewise, the absorption of the sulfonate (S=O) appears at 1140 cm^−1^ [45]. Based on these bands, the formation of the BL PAni is confirmed.

### 2.2. UV–Vis Study

#### 2.2.1. Optical Properties of Macro-RAFT, PAAMP-*b*-PVK, and BL PAni

**Figure 5:**
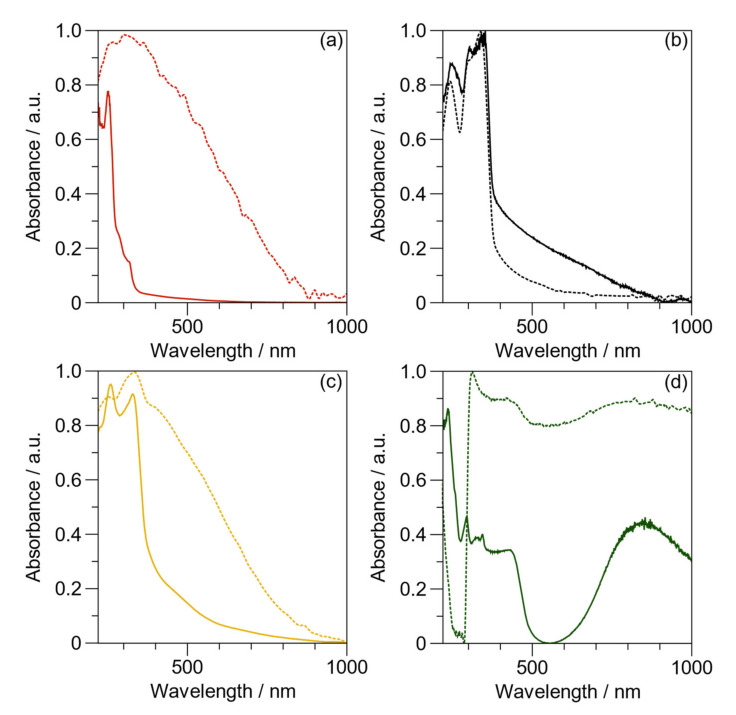
UV–Vis spectra of the samples in solution (solid line) and solid-state (dash line): (**a**) macro-RAFT; (**b**) poly(*N*-vinylcarbazole) (PVK); (**c**) PAAMP-*b*-PVK; (**d**) BL PAni.

Figure 5 shows the UV–Vis spectra of macro-RAFT in aqueous solution and in solid-state. In Figure 5a, displaying the results in aqueous solution, three absorption bands are observed at 250 nm, 284 nm, and 312 nm, corresponding to the n-π* transition of N^+^ quaternized nitrogen, the π-π* transition of anilinium ring [37], and the π-π* of C=S of CTA [46]. On the other hand, the spectrum in the solid-state has a broadband absorption between 230 and 800 nm, with a maximum at 301 nm. This finding could be related to the π-stacking interaction of anilinium ions [47].

The PVK (obtained by free radical polymerization in dimethyl sulfoxide) spectrum in water-dispersion, as seen in Figure 5b, displays the characteristic bands corresponding to the π-π* and n-π* transitions of carbazole heterocycle at 244, 302, and 337 nm [48]. In contrast, a similar pattern was obtained for the solid-state spectrum of PVK. Therefore, the water is not interacting with this hydrophobic polymer [48].

The spectrum of PAAMP-*b*-PVK in solution, as seen in Figure 5c, presents two main bands at 257 and 329 nm while in the solid state, these appear at 250 nm and 336 nm. Compared with PAAMP-*b*-PVK in dispersion, the spectrum of the block copolymer in the solid-state shows broadband absorption in the same visible region as macro-RAFT. Consequently, the copolymer block presents similar absorption spectra, indicating that the PVK segment size does not modify the optical properties.

The spectrum of the water-dispersed BL PAni, as seen in Figure 5d, exhibits the typical bands of PAni, i.e., the benzenoid band at 342 nm, the polaron band at 430 nm, and the bipolaron band (>700 nm) [49]. The spectrum also presents the typical bands of the carbazol heterocycle at 295 and 327 nm [48]. The solid-state spectrum of the BL PAni copolymer shows absorption throughout the visible range to the near-infrared region, except for the area from 230 to 300 nm. A possible explanation of this phenomenon is the formation of aromatic ring interactions between carbazole heterocycle and the polaron and bipolaron transitions of the PAni. Figure S5 presents the UV–Vis spectra of macro-RAFT, PVK, PAAMP-*b*-PVK copolymers, and BL PAni.

All synthesized polymers were studied by Raman spectroscopy with a laser at 244 nm (Figure S6). All Raman spectra display similar peaks, e.g., at ~1344 cm^−1^ and ~1615 cm^−1^, assigned to the γ(C-N) and γ(C=C) of the aromatic rings, respectively [50]. Hence, these findings confirm that all the polymers synthesized in the present work present electronic transitions at 244 nm.

#### 2.2.2. Dedoping Point of Poly(2-acrylamide-2-methyl-1-propanesulfonate-graft-polyaniline)-Block-Poly(*N*-vinylcarbazole)

**Figure 6:**
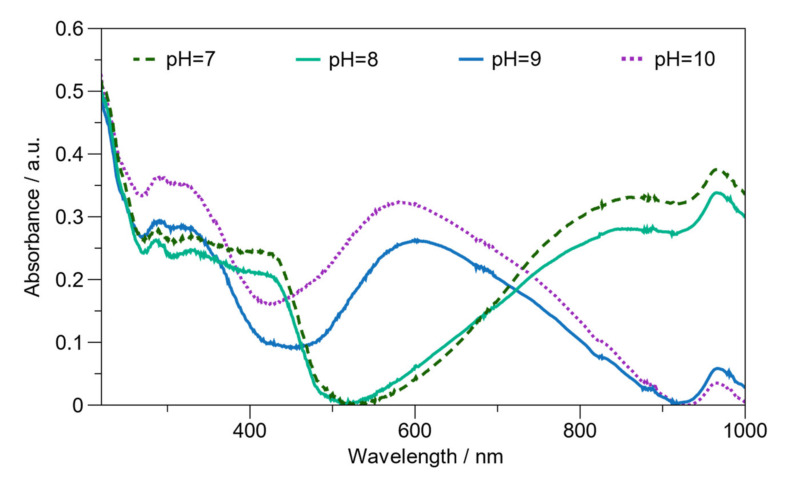
UV–Vis spectra of BL PAni, in water, and in the range where the doping point occurs from pH 7 to pH 10.

The dedoping point of BL PAni was studied by varying the pH from 1 to 13. Figure S7A illustrates the UV–Vis spectra from pH 1 to 13. The UV–Vis shows similar spectra below pH 6 and above pH 10. An abrupt change occurs between pH 8 and pH 9 owing to the dedoping point (Figure 6). At pH 6 and pH 7, it is observed that the intensity of bipolaron decreases until pH 8, whereas, at pH 9 and pH 10, the polaron broadband shows a blue shift, presenting a new band around ~600 nm characteristic of an emeraldine base structure [51]. In our previous work, the dedoping point of the PAni doping with AMPS manifested itself at pH 4.4 [37]. Similarly, the doping point of PAni utilizing sulfuric acid is 4.4 [52]. This phenomenon is due to the pKa of the aniline [37].

In contrast, the evidence does not establish a doping point between pH 4 and pH 5. This result can be explained by the formation of a π-stacking interaction between the aromatic rings of the carbazole group (PVK) and the benzenoid (PAni). This interaction regulates the doping point that PAni emeraldine reveals. Therefore, the result of BL PAni confirms that the π-stacking interaction plays an essential role in PAni stabilization. However, the overlapping of the electronic transitions of carbazole and the PAni polaron signal made it impossible to determine the dedoping point accurately. Consequently, the dedoping point of BL PAni is estimated between pH 8 and 9, i.e., the change from its conductive to the non-conductive structure.

#### 2.2.3. Optical Properties in Correlation with Temperature

**Figure 7:**
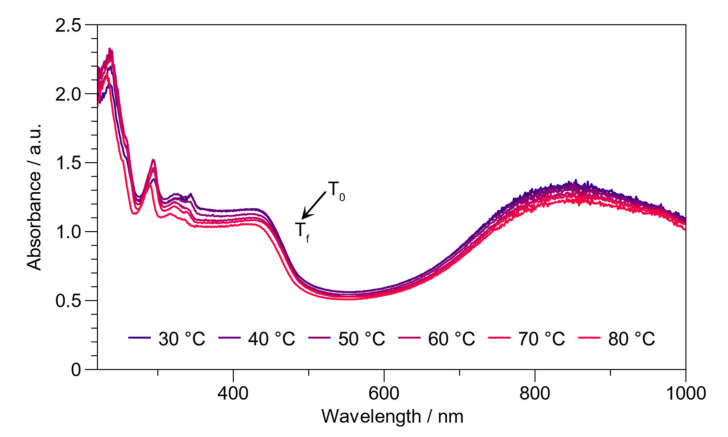
UV–Vis study of the BL PAni, dispersed in water, correlates with temperature from 30 to 80 °C.

The specific objective of this study was to evaluate π-stacking interactions concerning temperature (Figure 7). The effect of temperature on the optical properties from 30 to 80 °C was studied. As noted, the changes observed every 10 °C are insignificant, and even at 80 °C, the PAni preserves its characteristic bands corresponding to the polaron and bipolaron (Figure S7B). Commonly, PAni–HCl suffers dedoping at 70 °C [53]. Meanwhile, the spectrum of PAni H_2_SO_4_, at 80 °C, presents an undoped segment verifiable by the decreased bipolaron band and the blue shift (hypsochromic), as shown in Figure S7C. Therefore, BL PAni exhibits excellent optical properties compared to PAni H_2_SO_4_ at 80 °C.

### 2.3. Electroactive Properties of BL PAni

The electroactive properties determined by cyclic voltammetry were studied to complement the characterization of BL PAni. Sulfuric acid (1 M) and phosphate buffer (0.1 M) were employed as the electrolytes.

When sulfuric acid was used as an electrolyte, the voltammogram shows two oxidative peaks at 0.23 and 0.80 V (Figure S8A). These peaks correspond to the transitions of lecuoeraldine/emeraldine and emeraldine/pernigraniline oxidation states. PVK was also reported to exhibit an oxidation peak at 0.83 V [54]. Therefore, the signal close to 0.8 V corresponds to the transition of PAni emeraldine/pernigraniline, overlapping with the oxidation peak of PVK [24]. As noted, the current at 0.23 and 0.80 V diminishes with the number of cycles, and a new peak appears at 0.47 V. Possible explanations for the first phenomenon may be attributed to the depolymerization of PAni [55], the oxidative electropolymerization of PVK, or the crosslinking of carbazole rings [54,56]. An alternative explanation of these findings (broadband at 0.47 V) is that fragments of depolymerized PAni could oxidate with carbazole rings creating a new conjugated chain [57].

**Figure 8:**
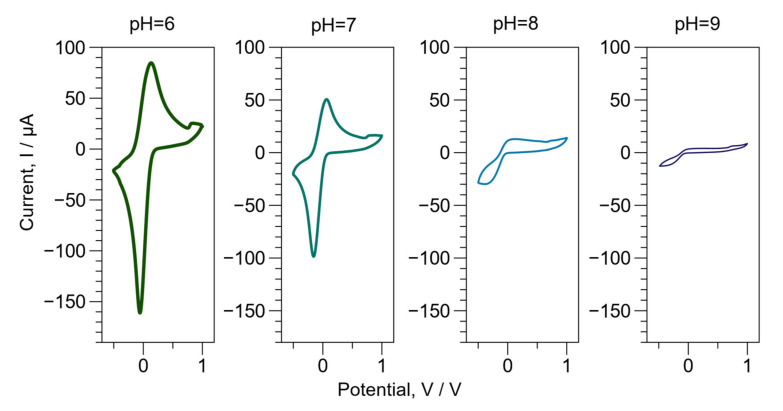
Electroactivity properties as a function of pH (6 to 9) by cyclic voltammetry of the BL PAni.

An evaluation of the electrochemical properties regarding pHs from 6 to 9 was conducted (Figure 8). In addition, Figure S8B–E displays cyclic voltammetry in correlation with pH, with 10 cycles. The pH of the buffer phosphates was adjusted with NaOH. The voltammograms only present the transition from leucoemeralidine/emeraldine due to the low ionic strength of the buffer phosphates at these pHs [58]. When the pH increases, it is important to note that the voltammogram signals decrease and shift to lower potentials, indicating the progressive dedoping of PAni [4]. It was also evident that the BL PAni presents a decreasing electroactivity in correlation with pH. The advantage of BL PAni is that the interaction between PAni and PVK provides pH stability due to the π-stacking interaction. Furthermore, it improves and solves the problems presented by PAni-based biosensors [59].

### 2.4. Thermal Stability

#### 2.4.1. Thermogravimetric Analysis

**Figure 9:**
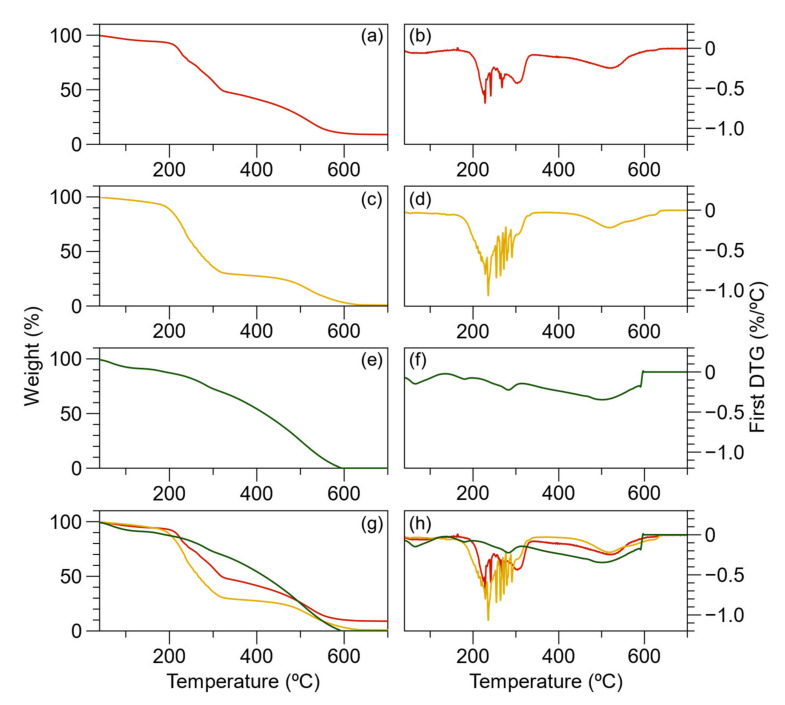
(**a**) Thermogram and (**b**) First derivative (DTG) of macro-RAFT; (**c**) Thermogram and (**d**) DTG of PAAMP-*b*-PVK; (**e**) Thermogram and (**f**) DTG of PAAMP-*b*-PVK; (**g**) Thermogram and (**h**) DTG of macro-RAFT (red), PAAMP-*b*-PVK (yellow), and BL PAni (green).

Because BL PAni was synthesized from a sequence of polymerizations, it is vital to understand the decomposition and degradation of each polymerization by TGA. Furthermore, BL PAni exhibited excellent optical properties at 80 °C when evaluated with UV–Vis. Figure 9 illustrates the weight loss thermograms and first derivatives (DTG) of the macro-RAFT, PAAMP-*b*-PVK, and BL PAni. Temperature assignment was performed using DTG. The thermogram of the macro-RAFT has four stages of thermal degradation, which are assigned to solvent loss (80 °C), anilinium ion loss (228 °C) [60], desulfonation (302 °C) [61], and polymer backbone degradation (523 °C) [62]. The decomposition of the anilinium ion and desulfonation of the macro-RAFT represents a weight loss of 48%. It is well known that sulfonated organic compounds heated in an oxygen atmosphere produce sulfate salts whose decomposition of 10% is above 700 °C [61]. This stage has a theoretical weight loss of 58%, which coincides with the experimental thermograms. At a maximum temperature of 523 °C, thermal degradations of the main polymer chain occur. The theoretical weight loss is 42%, which corresponds to the value of the weight loss thermogram [62].

The PAAMP-*b*-PVK copolymer presents three stages associated with the decomposition and thermal degradation of polymers. The first stage occurs below 150 °C due to volatile substances with a weight loss of 8%. The next stage is polymer decomposition (150–340 °C) which may be extremely complex, and many reactions are involved simultaneously. The TG curves in air atmosphere display a DTG peak with several overlapping parts. The decomposition reactions of anilinium ions (228 °C) [60], desulfonation (302 °C) [61], and the carbazole group occur at this stage with a weight loss of 73%. The theoretical weight loss of PAAMP-*b*-PVK(C) is 13.8%, 12.8%, and 46% for the decomposition of anilinium ions, desulfonation, and carbazole groups, respectively.

Moreover, the degradation of the copolymer main chain appears at 534 °C. It is worth saying that the carbazole rings can be oxidated with anilinium ions forming a new bond [57]. The thermal degradation of the poly(AMPS) segment—the second stage—appears near 534 °C. As observed in Figure S9, the PAAMP-*b*-PVK(A), PAAMP-*b*-PVK(B), and PAAMP-*b*-PVK(C) thermograms are similar, the copolymer constitution notwithstanding.

The thermogram of BL PAni illustrates four stages of thermal degradation. A weight loss of 10% due to volatiles is exhibited at a maximum temperature of 78 °C. In comparison with the PAAMP-*b*-PVK(C) thermogram, the result is utterly different in the region from 150 to 340 °C. The second stage, at 178 °C, was attributed to the loss of the doping agent of PAni. The third stage, at 283 °C, corresponds to the degradation of the PVK main chain that does not have a π-stacking interaction with PAni [57]. Additionally, the last transition, near 500 °C, is ascribed to the degradation of the AMPS-*g*-PAni segment [62,63]. This final transition accounts for 70% of the sample weight loss.

Based on the formulation of BL PAni, the theoretical composition by weight is 13%, 22%, and 65% for hydrogen sulfate (doping), emeraldine salt polyaniline, and PAAMP-*b*-PVK(C), respectively.

#### 2.4.2. Differential Scanning Calorimetry

**Figure 10:**
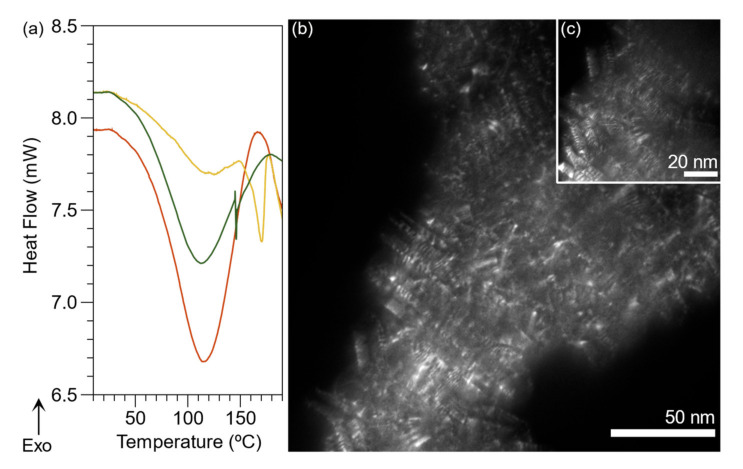
(**a**) DSC thermogram of macro-RAFT (red); PAAMP-*b*-PVK (yellow); BL PAni (green). (**b**,**c**) TEM micrograph of BL PAni.

TGA was complemented with DSC for the macro-RAFT, PAAMP-*b*-PVK, and BL PAni, as seen in Figure 10 and Figure S10. The macro-RAFT shows only an endothermic peak at 134 °C, suggesting a crystalline polymer due to the formation of the graft point generating a physical interaction between the anilinium ions. In our previous study, the poly(anilinium 2-acrylamide-2-methyl-1-propanesulfonate) exhibited a glass transition temperature of 109 °C; even so, this was obtained from an altogether different methodology [37].

Additionally, the endothermic peaks of PAAMP-b-PVK(C) are revealed at 112 °C and 176 °C assigned to the PAAMP and PVK segments, respectively. A possible explanation is that the π-stacking interaction between anilinium ions and carbazole provides crystalline regions. Therefore, the block copolymer has two endothermic peaks assigned to the different segments of macro-RAFT and PVK. The presence of the two peaks is consistent with a block copolymer structure.

The BL PAni presents only one endothermic peak at 114 °C. The conventional systems with PAni grafts have copolymer main chain transitions and an exothermic peak at 250 °C corresponding to polymer degradation [64,65]. The BL PAni commences presenting decomposition from 178 °C and degradation to 283 °C, according to the results of the TGA. The fact that BL PAni only provides an endothermic transition may be due to the interactions between the three segments, which are associated with the typical BBC crystal structure [66]. Shen et al. reported a similar phenomenon attributed to the interaction between the carbazol substituent (3-(*N*-carbazolyl)propyl acrylate) and the conjugated main chain of polythiophene [67]. Therefore, our findings can be explained due to the interaction between carbazole and PAni.

Moreover, the confirmation of a crystalline polymer from BL PAni was performed by TEM (Figure 10b,c). The formation of lamellae characterizes the crystalline region. Dark field micrographs show this pattern; however, the crystalline unit is not yet defined [68].

## 3. Materials and Methods

### 3.1. Materials

Aniline (Sigma-Aldrich, St. Louis, MO, USA, >99.5%) was vacuum distilled prior to use, while 2-acrylamide-2-methylpropane-1-sulfonic acid (Sigma-Aldrich, St. Louis, MO, USA, >99%), *N*-vinylcarbazole (Merk, Sigma-Aldrich, St. Louis, MO, USA, >98%), 4,4′-azobis(4-cyanopentanoic acid) (ACVA) (Sigma-Aldrich, St. Louis, MO, USA, >98%), 4-cyano-4-(phenylcarbonothioylthio) pentanoic acid (CTA) (Sigma-Aldrich, St. Louis, MO, USA, >99%), toluene (Sigma-Aldrich, St. Louis, MO, USA, >98%), hexanol (Merk, Sigma-Aldrich, St. Louis, MO, USA >98%), hydrochloric acid (Sigma-Aldrich, St. Louis, MO, USA, >37%), sulfuric acid (Sigma-Aldrich, St. Louis, MO, USA, >98%), sodium dibasic phosphate (Sigma-Aldrich, St. Louis, MO, USA, >99%), sodium hydroxide (Sigma-Aldrich, St. Louis, MO, USA, >98%), deuterium oxide (Sigma-Aldrich, St. Louis, MO, USA, 99.9%), and deuterated tetrahydrofuran (Sigma-Aldrich, St. Louis, MO, USA, >99.9%) were used as received.

### 3.2. Poly(anilinium 2-acrylamide-2-methyl-1-propanesulfonate), Macro-RAFT

All experiments were conducted in a three-necked reactor equipped with magnetic agitation, temperature control, and an inert atmosphere. A solution of the monomer 2-acrylamide-2-methyl-1-propanesulfonic acid (9.65 mmol), CTA 4-cyano-4-(phenylcarbonothioylthio)pentanoic acid (0.193 mmol), 4,4′-azobis(4-cyanopentanoic acid) (ACVA) (0.048 mmol), and 18 mL of tri-distilled water was prepared. The [monomer]:[CTA]:[initiator] ratio was 50:1:0.25. The solution was bubbled with argon for 30 min. The flask three-necked reactor was then heated to 70 °C for six hours with constant agitation. At the end of the polymerization, functionalization was performed at a temperature of 25 °C. The monomer aniline (17.459 mmol) was slowly added to the reactor and mixed for four hours. The macro-RAFT was precipitated with acetone (non-solvent) and removed aniline without functionalization. ^1^H NMR (400 MHz, D_2_O) δ 7.70–6.88 (m, 5H), 3.21 (d, J = 27.7 Hz, 2H), 2.20–2.10 (m, 2H), 1.91 (d, J = 61.1 Hz, 1H), 1.42–1.25 (m, 6H). ^13^C NMR (101 MHz, D_2_O) δ 212.54, 130.15, 129.99, 129.11, 124.95, 122.85, 30.76, 30.21, 26.41, 23.54. FT-IR (ATR) vmax/cm^−1^ 3417, 3304, 2621, 1641, 1625, 1600, 1494, 1280–1150, 1034.

### 3.3. Poly(anilinium 2-acrylamide-2-methyl-1-propanesulfonate)-Block-Poly(N-vinylcarbazole), PAAMP-b-PVK

The synthesis was conducted in a three-necked reactor equipped with magnetic agitation, temperature control, and an inert atmosphere. First, the monomer *N*-vinylcarbazole (0.833 mmol or 1.665 mmol (for example) or 2.498 mmol) was dissolved into toluene (1 mL) and placed in the reactor. Subsequently, a solution of macro-RAFT (500 mg) in tri-distilled water (7 mL) and hexanol (2 mL) was added. Then, the mixture was sonicated for 30 min under an inert atmosphere. The reactor was then heated to 80 °C. The initiator ACVA (5 mg) dispersed in water (1 mL) was added at this temperature. The copolymerization was left for 12 h with continuous magnetic agitation. Afterward, the reactor was left to cool at room temperature and mixed with acetone (50 mL). The copolymer solution was centrifuged for 10 min at 6000 rpm. The precipitate contained the by-products and reagents that did not react. The liquid supernatant contained the copolymer, which was separated and evaporated at room temperature.

^1^H NMR (400 MHz, deuterium oxide) δ 8.16 (m = 7.7 Hz, 3H), 7.89–7.55 (m, 5H), 7.51 (t, J = 8.2, 7.0, 1.2 Hz, 2H), 7.32–7.20 (t, 2H), 3.44 (s = 40.5 Hz, 2H), 2.34 (m = 27.9 Hz, 2H), 2.08–1.08 (m, 10H). ^13^C NMR (101 MHz, D_2_O) δ 226.13, 140.16, 130.13, 128.91, 125.32, 123.21, 122.87, 119.87, 119.54, 118.35, 110.86, 39.13, 34.02, 30.21, 26.55. FT-IR (ATR) vmax/cm^−1^ 3050, 2920, 2624, 1660, 1600, 1450, 1177.

### 3.4. Poly(2-acrylamide-2-methyl-1-propanesulfonate)-Block-Poly(N-vinylcarbazole)/Graft-Polyaniline, BL PAni

The brush-like polyaniline (BL PAni) was prepared by interfacial oxidative polymerization. With this technique, polymerization occurs between two immiscible liquids. The solvent solution contains aniline (0.214 mmol) in dichloromethane (2 mL), and the aqueous solution (5 mL) is constituted by PAAMP-*b*-PVK [1.0:1.5] (60 mg), ammonium persulfate (0.4 mmol), and sulfuric acid (200 µL). The solvent solution was carefully placed in the aqueous solution, creating a two-phase system. The polymerization proceeded for 24 h at 4 °C. Subsequently, the dichloromethane was extracted, and the aqueous phase was centrifuged for 10 min at 6000 rpm. The product was obtained by removing the supernatant. The removal of polymerization by-products was carried out with three cycles of redispersion and centrifugation. A mixture of ethanol and sulfuric acid (10:1) was used for redispersion. The copolymer was dried at room temperature. FT-IR (ATR) vmax/cm^−1^ 3230, 3055, 1650, 1582, 1483, 1288, 1140.

### 3.5. Characterization

The soluble polymers were studied by proton and carbon nuclear magnetic resonance (^1^H and ^13^C NMR), respectively, using a spectrometer (NMR Bruker Ascend 400 MHz, Billerica, MA, USA) at 400 MHz, 7.05 T, and 25 °C. The solvent used was deuterium oxide for the macro-RAFT sample and a mix of deuterium oxide and deuterated tetrahydrofuran (70:30) for the block copolymers samples. In addition, an infrared spectrometer equipped with an ATR accessory (GX-FT-IR, PerkinElmer, Waltham, MA, USA) was employed to complement the structural elucidation. The spectra were acquired with a spectral window of 4000 to 500 cm^−1^, with a resolution of 4 cm^−1^, and achieved 30 scans.

The optical properties of the polymers were studied in solution and solid-state (integration sphere) by UV–Vis spectroscopy (Evolution 220, Thermo Fisher Scientific, Waltham, MA, USA).

In a three-electrode system, the electrochemical properties were studied by cyclic voltammetry using a potentiostat (Emstat 3+ blue, PalmSense BV, Houten, The Netherlands). An Ag/AgCl electrode was used as the reference electrode, the counter electrode was a platinum plate (1 cm^2^), and a glassy electrode containing a polymer sample was the working electrode. The electrolytes utilized in this technique were sulfuric acid (1 M) and a buffer phosphate (0.1 M) adjusted with NaOH. The potential window of cyclic voltammetry was from −0.5 to 1.0.

The thermal stability of the polymers was determined by thermogravimetric analysis (SDT Q600, TA Instruments, New Castle, DE, USA). The temperature range studied was from room temperature (27 °C) to 700 °C, at a heating rate of 10 °C min^−1^ under an air atmosphere. The endothermic peaks regarding macro-CTA and PVK were determined using a differential scanning calorimeter (DSC Q2000, TA Instrument, New Castle, DE, USA) utilizing a 10 mg sample. The temperature range analyzed was from room temperature to 250 °C at the rate of 5 °C min^−1^ under an argon atmosphere.

The morphological characterization of BL PAni was performed using transmission electron microscopy (TEM Hitachi 7700, Tokyo, Japan) operated at 100 kV. The BL PAni sample dispersed in water was placed on the surface of a lacey formvar-carbon supported copper grid, size 200 mesh.

## 4. Conclusions

In the present study, brush-like polyaniline (BL PAni) was synthetized from a block copolymer containing a PVK segment, which exhibits electroactive properties at a neutral pH and optical properties throughout the visible range to the near-infrared and is optically stable up to 80 °C. Furthermore, the findings show that the formation of π-stacking interactions between polyaniline (PAni) and poly(*N*-vinylcarbazole) (PVK) favored these properties. Notably, the properties of BL PAni are remarkable since it is also slightly water soluble, reducing the limitation imposed by conventional PAni emeraldine salt. Contrary to the reported PAni graft, BL PAni shows a change in the doping point between pH 8 and pH 9, and therefore exhibits excellent electroactive and optical properties at a neutral pH.

Moreover, the BL PAni properties are attractive for the development of electrochemical biosensors due to the system holding its electroactivity at a neutral pH. BL PAni could be employed as a transducer active at a physiological pH. In addition, the BL PAni macrostructure could be vital for the easy support of metallic nanoparticles or enzymes to develop different kinds of biosensors. Additionally, it will be interesting to study the photoexcitation properties of the system to consider the possible application in OLED development due to their wide absorption range, which could be employed in solar cell design.

It is worth mentioning that the sequences of polymerizations employed to develop BL PAni allow for the regulation of the dedoping point, and the optical and electroactivities properties, which could emphasize some intrinsic properties of the PAni such as conductivity, polarity, plasticity, to mention some; therefore, PAni properties could be tailored.

## Figures and Tables

**Table 1:** Formulations of the poly(anilinium 2-acrylamide-2-methyl-1-propanesulfonate)-*b*-poly(*N*-vinylcarbazole) (PAAMP-*b*-PVK) via RAFT-mediated surfactant-free emulsion polymerization.

Copolymers	NVK (g)	Macro-RAFT (mg)	Initiator * (mg)	Toluene (mL)	Water (mL)	Hexanol (mL)	Yield (%)
PAAMP-*b*-PVK(A)	0.16	500	5	1	7	2	82
PAAMP-*b*-PVK(B)	0.32	500	5	1	7	2	75
PAAMP-*b*-PVK(C)	0.48	500	5	1	7	2	79

* ACVA, 4,4′-azobis(4-cyanopentanoic acid).

**Table 2:** Proton integration (int.) of H_1_ and H_c_ for molecular weight calculation of the PAAMP-*b*-PVK.

Copolymers	Int. H_1_	Poly(Ani-AMPS) DP	Int. Hc	PVK DP	PVK, Mn (g/mol)	Mn (g/mol) *
PAAMP-*b*-PVK(A)	2	89	0.75	33	6449	32,358
PAAMP-*b*-PVK(B)	2	89	2.11	94	18,166	44,147
PAAMP-*b*-PVK(C)	2	89	3.25	145	28,021	54,002

* Molecular weight of the block copolymer.

**Table 3:** Comparison of theoretical molecular weight (Mn) versus Mn calculated using NMR.

Copolymers	[NVK]:[Macro-RAFT]:[I]	Yield (%)	Mn (Theoretical) (g/mol)	Mn (NMR) (g/mol)
PAAMP-*b*-PVK(A)	41.5:1:0.9	80	32,396	32,358
PAAMP-*b*-PVK(B)	83.0:1:0.9	75	38,010	44,147
PAAMP-*b*-PVK(C)	124.5:1:0.9	79	44,987	54,002

## Data Availability

Not applicable.

## Supplementary Materials

The following supporting information can be downloaded at: https://www.mdpi.com/article/10.3390/ijms23158085/s1.
