# Supplementary material for: Brush-like Polyaniline with Optical and Electroactive Properties at Neutral pH and High Temperature
Source: Int J Mol Sci. 2022 Jul 22;23(15):8085. doi: 10.3390/ijms23158085 (PMC9330365; doi:10.3390/ijms23158085)
Supplement: Supplementary file 1 [file ijms-23-08085-s001.zip › ijms-1784844-supplementary.pdf]

Brush-like PAni with optical and electroactivity properties at neutral pH.

Alain Salvador Conejo-Dávila [1], Carlos Rafael Casas-Soto [1], Eider Pedro Aparicio-Martínez[1], David Chávez-Flores[2], Víctor Hugo Ramos-Sánchez[2], Rocio Berenice Dominguez[3], Velia Carolina Osuna[3], Anayansi Estrada-Monje[4], Alejandro Vega-Rios[1*], and E. Armando Zaragoza-Contreras [1*].

1 Department of Engineering and Materials Chemistry, Centro de Investigación en Materiales Avanzados, S.C., Miguel de Cervantes No.120, Complejo Industrial Chihuhua, Chihuahua C.P.31136, México; alain.conejo@cimav.edu.mx (A.S.C.-D.); carlos.casas@cimav.edu.mx(C.R.C.-S.) eider.aparicio@cimav.edu.mx (E.A.-M.).

2 Facultad de Ciencias Químicas, Universidad Autónoma de Chihuahua, Chihuahua CP.31125, México; dchavezf@uach.mx (D.C.-F.) vramos@uach.mx (V. H. R. S.).

3 CONACyT-CIMAV S.C., Miguel de Cervantes 120, Complejo Industrial Chihuahua, Chihuahua CP.31136, México; berenice.dominguez@cimav.edu.mx (R.B.D.).

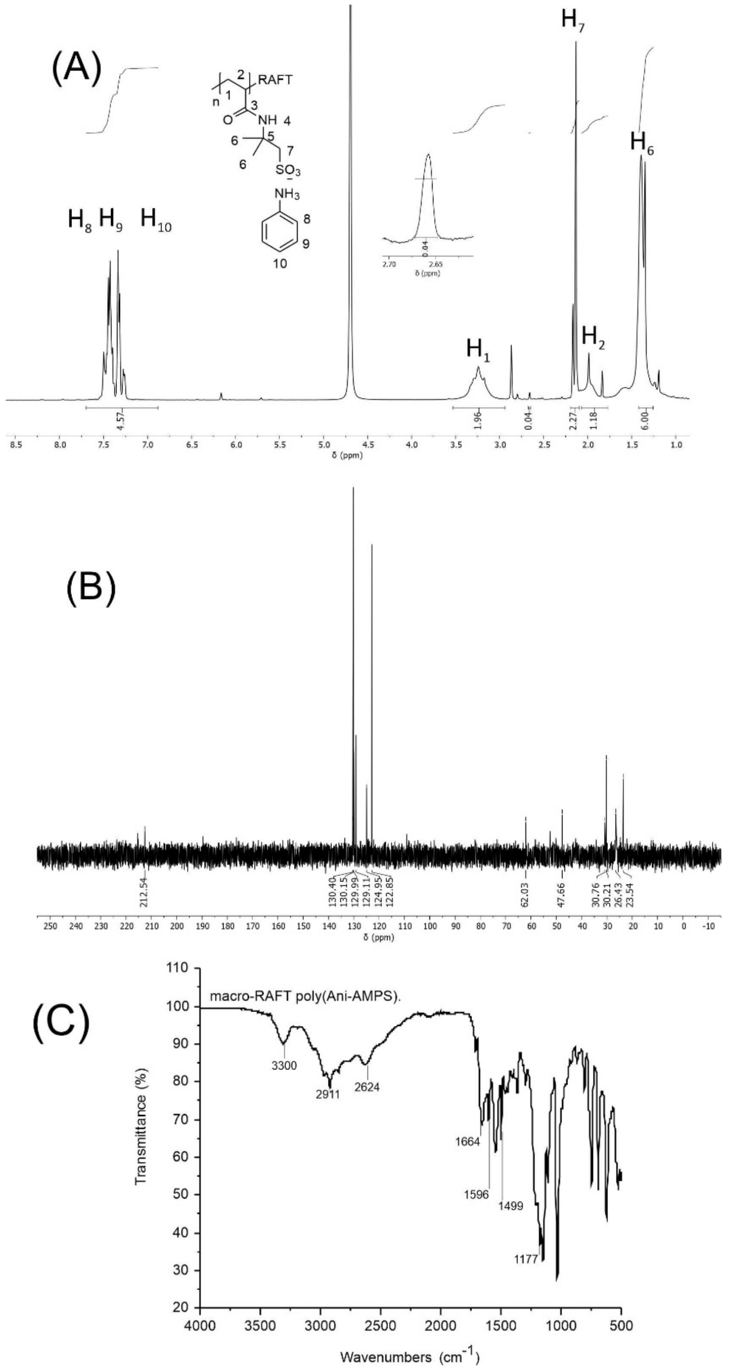

Figure S1. ^{1}H NMR, ^{13}C NMR, and FT-IR spectra of poly(anilinium 2-acrylamide-2-methyl-1-propanesulfonate) (macro-RAFT). (A) ^{1}H NMR (400 MHz, D_2O) δ 7.70 – 6.88 (m, 5H), 3.21 (d, J = 27.7 Hz, 2H), 2.20 – 2.10 (m, 2H), 1.91 (d, J = 61.1 Hz, 1H), 1.42 – 1.25 (m, 6H)., (B) ^{13}C NMR (101 MHz, D_2O) δ 212.54, 130.15, 129.99, 129.11, 124.95, 122.85, 30.76, 30.21, 26.41, 23.54 and (C) FT-IR of the macro-RAFT.

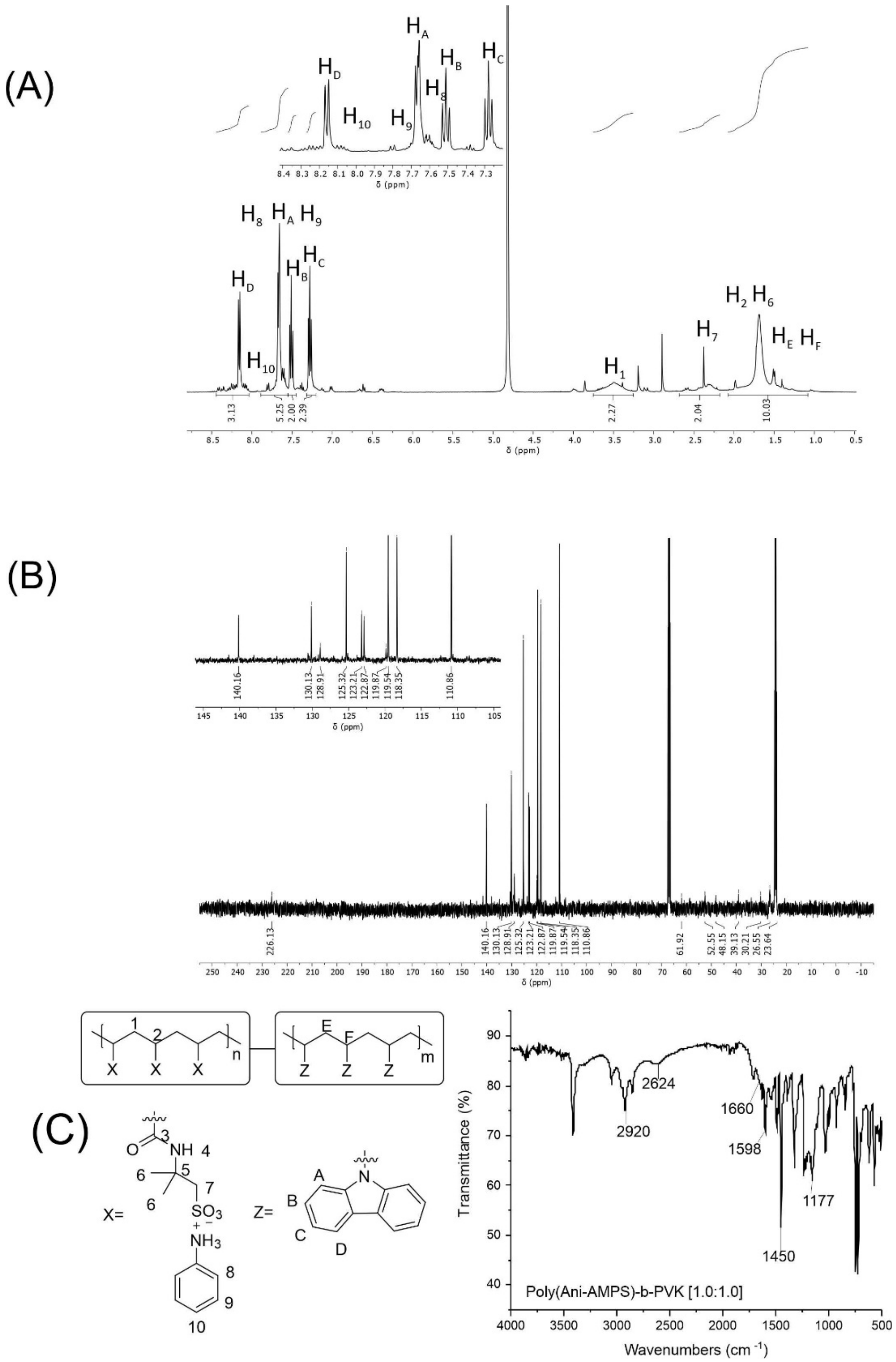

Figure S2. ^{1}H NMR, ^{13}C NMR, and FT-IR spectra of poly(anilinium 2-acrylamide-2-methyl1-propanesulfonate)-*b*-poly(*N*-vinylcarbazole) [PAAMP-*b*-PVK (B)] (A) ^{1}H NMR (400 MHz, Deuterium Oxide) δ 8.16 (m = 7.7 Hz, 3H), 7.89 – 7.55 (m, 5H), 7.51 (t, J = 8.2, 7.0, 1.2 Hz, 2H), 7.32 – 7.20 (t, 2H), 3.44 (s = 40.5 Hz, 2H), 2.34 (m = 27.9 Hz, 2H), 2.08 – 1.08 (m, 10H). (B) ^{13}C NMR (101 MHz, D_2O) δ 226.13, 140.16, 130.13, 128.91, 125.32, 123.21, 122.87, 119.87, 119.54, 118.35, 110.86, 39.13, 34.02, 30.21, 26.55; (C) FT-IR of the PAAMP-*b*-PVK (B).

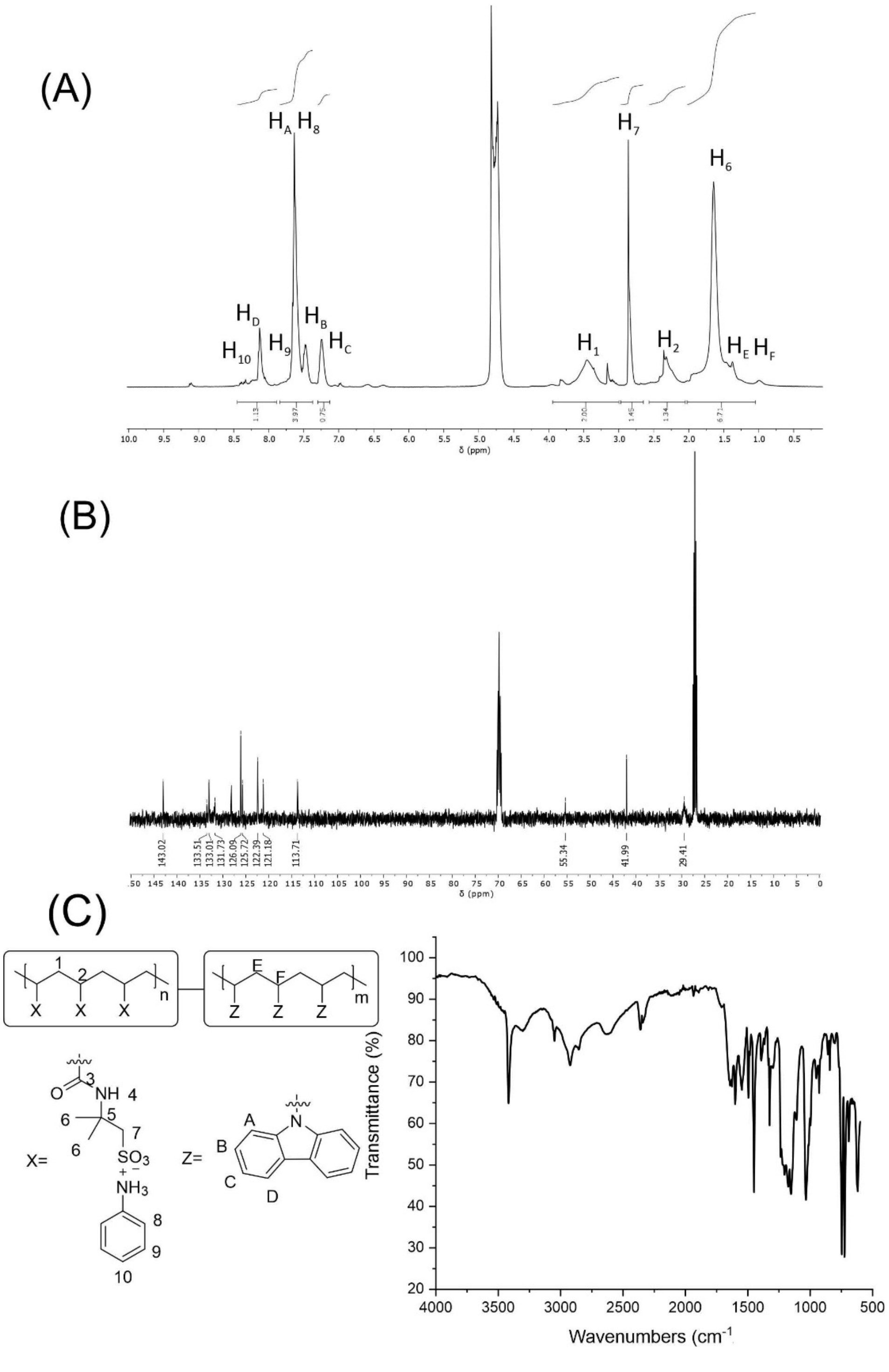

Figure S3. ^{1}H NMR, ^{13}C NMR, and FT-IR spectra of poly(anilinium 2-acrylamide-2-methyl-1-propanesulfonate)-*b*-poly(*N*-vinylcarbazole) [PAAMP-*b*-PVK (A)] (A)^{1}H NMR (400 MHz, Deuterium Oxide) δ 8.45 – 7.89 (m, 1H), 7.84 – 7.37 (m, 4H), 7.30 – 7.13 (m, 1H), 3.95 – 3.00 (m, 2H), 2.97 – 2.65 (m, 1H), 2.57 – 2.05 (m, 1H), 1.64 (s, 7H). (B) ^{13}C NMR (101 MHz, D_2O) δ 143.02, 133.51, 133.01, 131.73, 126.09, 125.72, 122.39, 121.18, 113.71, 55.34, 41.99, 29.41; (C) FT-IR of the PAAMP-*b*-PVK (A).

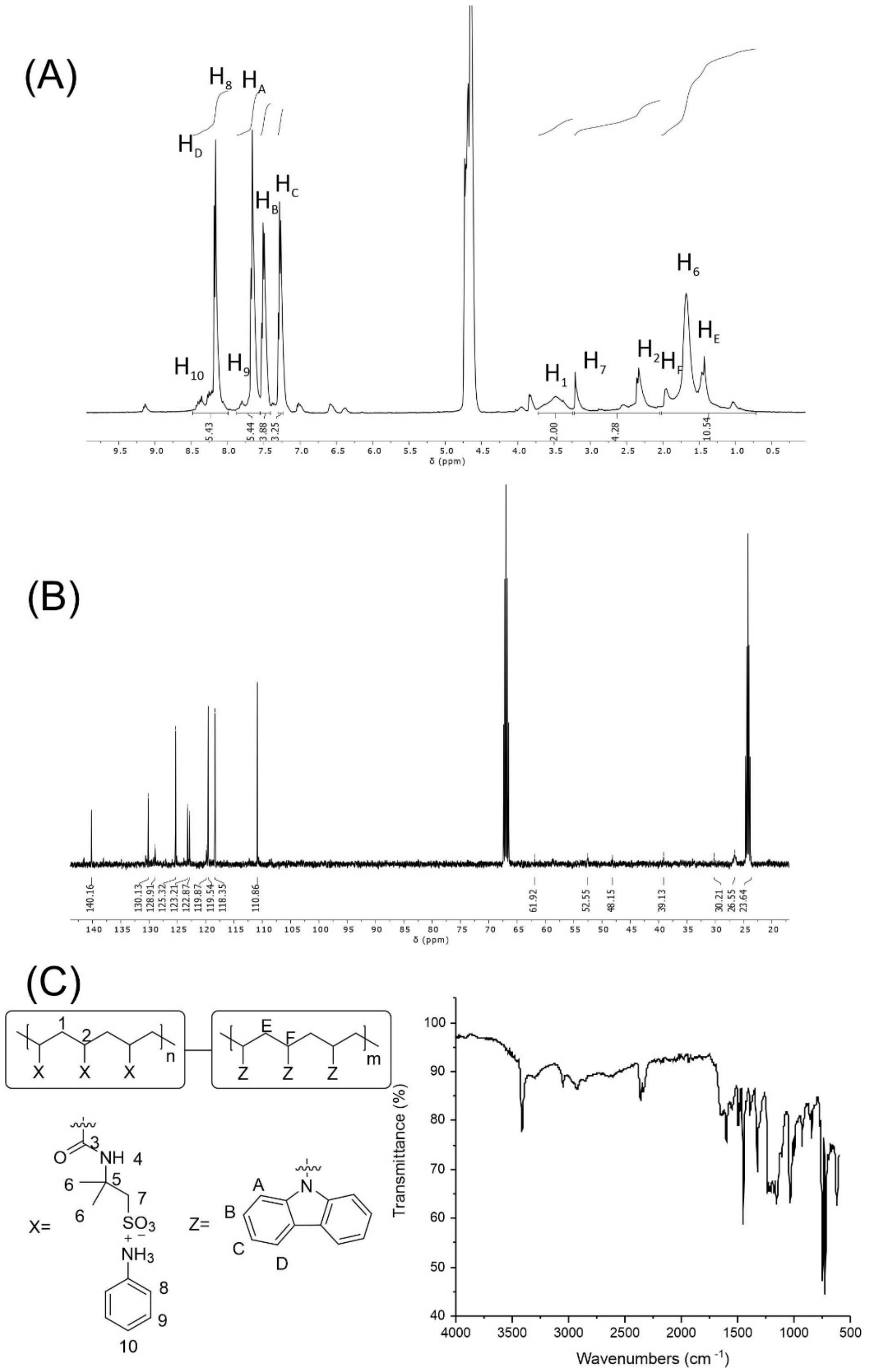

Figure S4. ^{1}H NMR, ^{13}C NMR, and FT-IR spectra of poly(anilinium 2-acrylamide-2-methyl-1-propanesulfonate)-*b*-poly(*N*-vinylcarbazole) [PAAMP-*b*-PVK (C)] (A) ^{1}H NMR (400 MHz, Deuterium Oxide) δ 8.48 – 7.99 (m, 5H), 7.66 (t, J = 7.3 Hz, 5H), 7.50 (q, J = 7.4 Hz, 4H), 7.26 (qd, J = 10.5, 6.6, 6.0 Hz, 4H), 3.43 (d, J = 41.0 Hz, 2H), 2.86 – 2.07 (m, 3H), 2.02 – 0.95 (m, 10H). (B) ^{13}C NMR (101 MHz, D_2O) δ 226.13, 140.16, 130.13, 128.91, 125.32, 123.21, 122.87, 119.87, 119.54, 118.35, 110.86, 39.13, 34.02, 30.21, 26.55; (C) FT-IR of PAAMP-*b*-PVK (C).

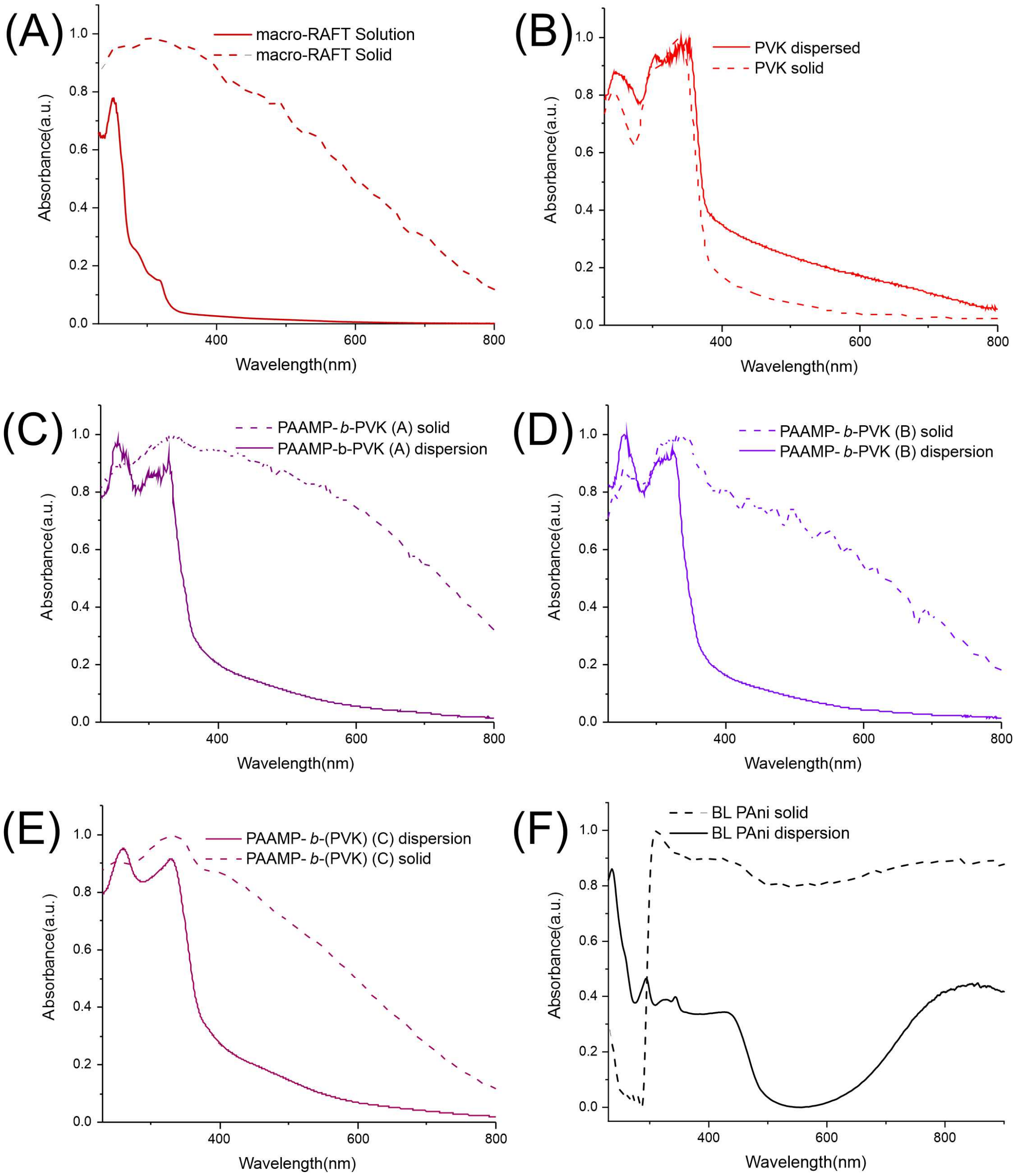

Figure S5. UV-vis spectra. (A) macro-RAFT, (B) PVK, (C) PAAMP-*b*-PVK (A), (D) PAAMP-*b*-PVK (B), (E) PAAMP-*b*-PVK (C), (F) BL PAni.

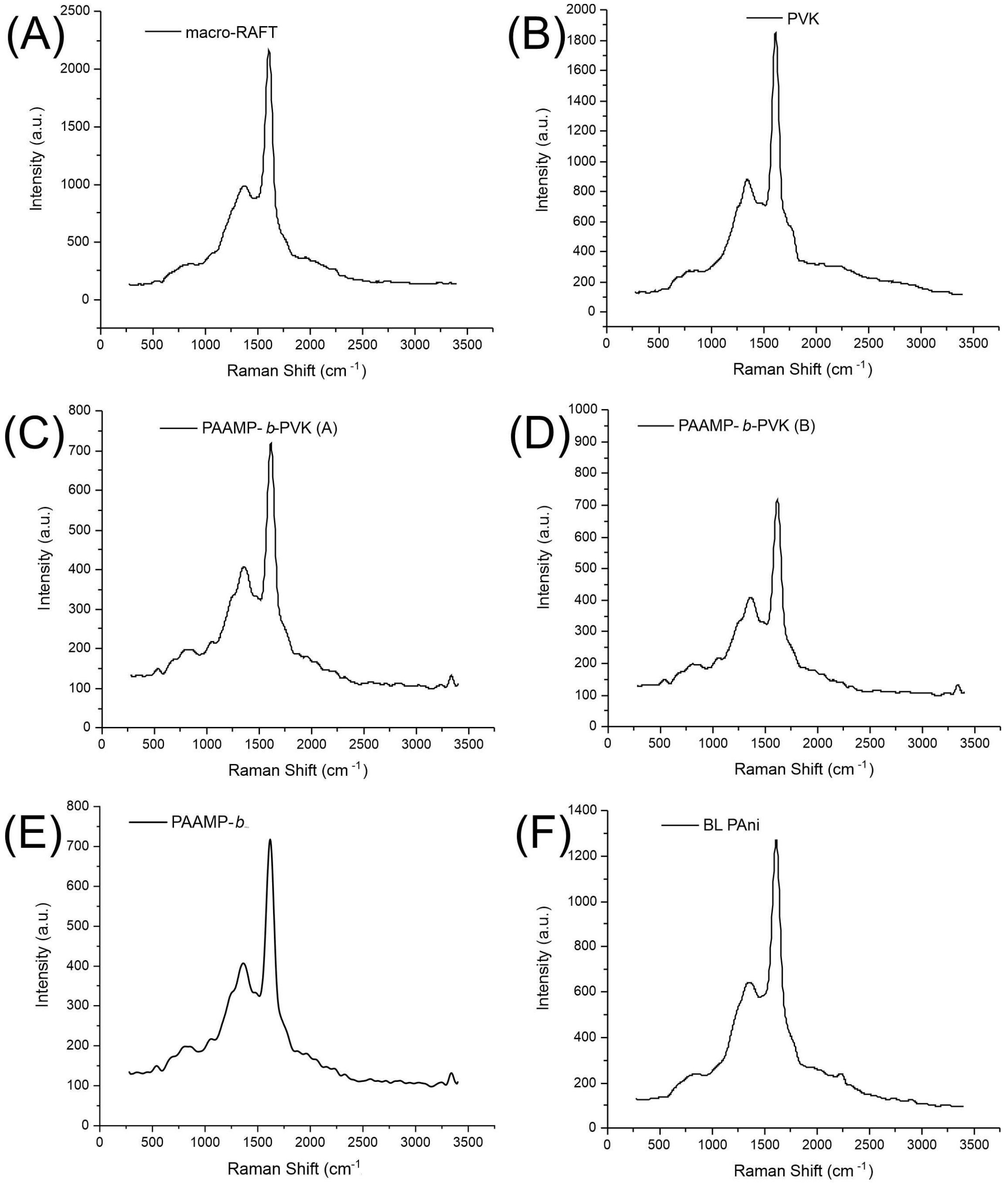

Figure S6. Raman spectra (wavelength=244 nm). (A) macro-RAFT, (B) PVK, (C) PAAMP-*b*-PVK(A), (D) PAAMP-*b*-PVK (B), (E) PAAMP-*b*-PVK (C), (F) BL PAni.

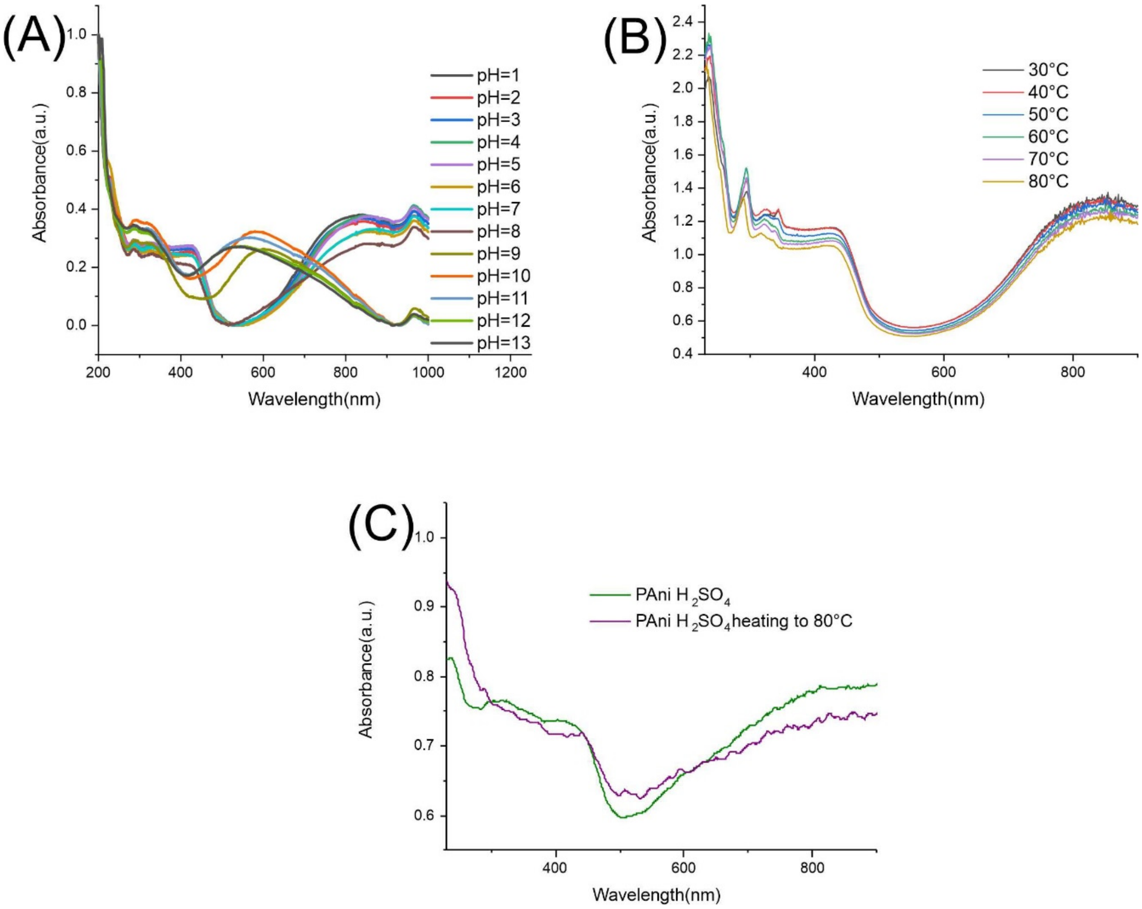

Figure S7. UV-vis spectra. (A) BL PAni at different pH from 1 to 13, (B) BL PAni at different temperatures from 30°C to 80°C, (C) PAni of H_2SO_4 at 30°C and 80°C,

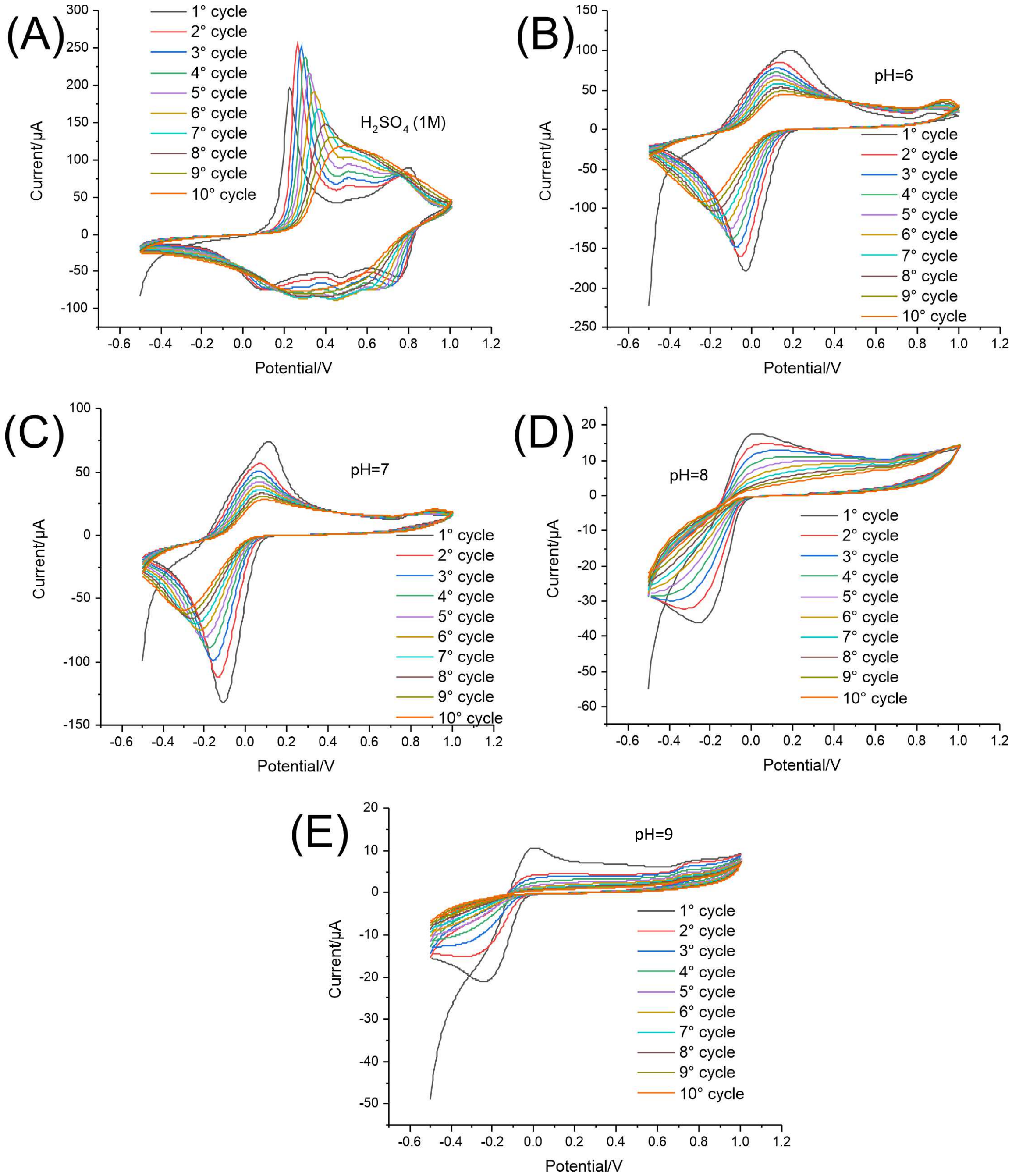

Figure S8. Voltammograms of BL PAni. (A) H_2SO_4 (1M); (B) pH=6 (buffer phosphates electrolyte); (C) pH=7 (buffer phosphates electrolyte); (D) pH=8 (buffer phosphates electrolyte); (E) pH=9 (buffer phosphates electrolyte).

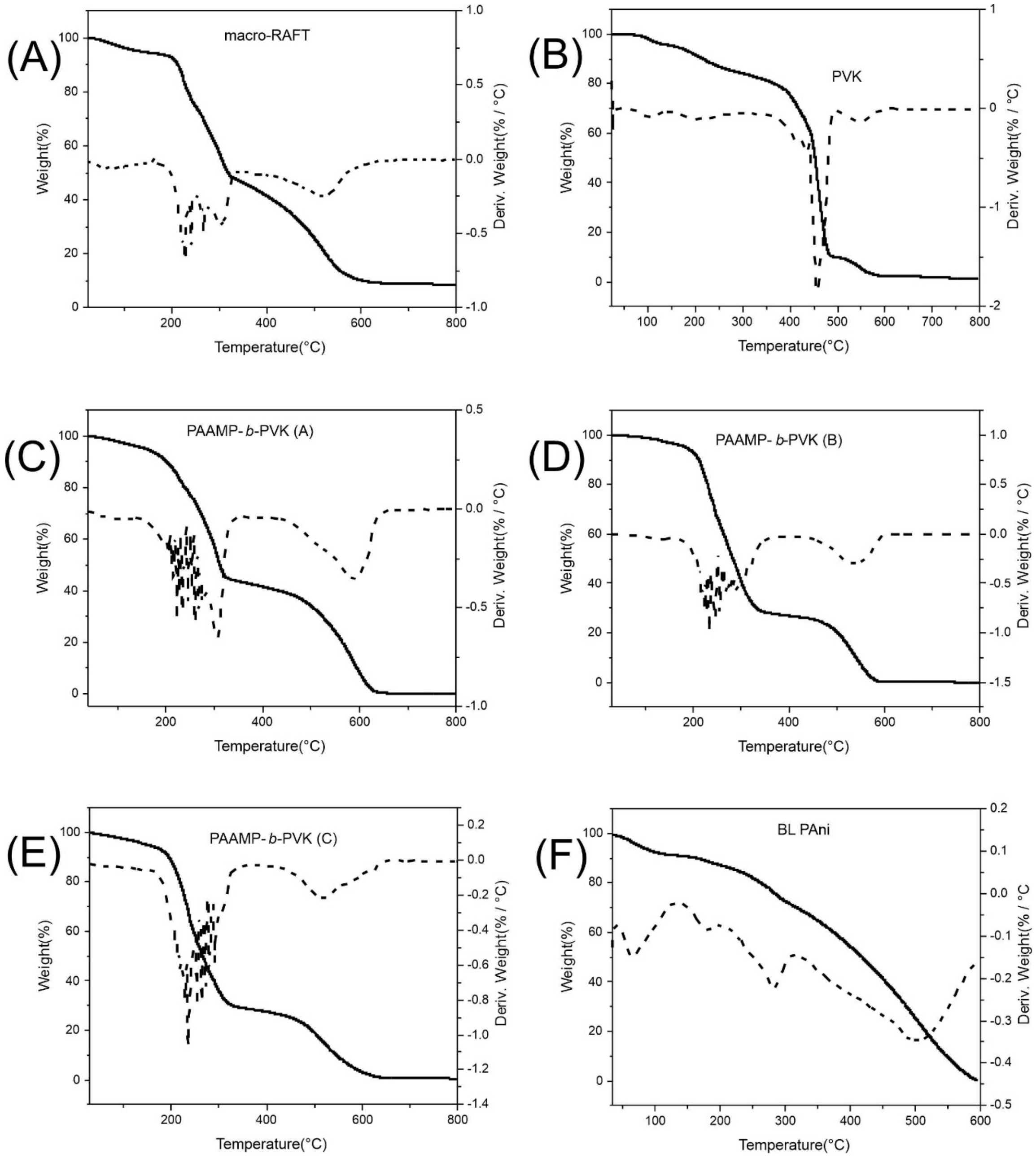

Figure S9. Thermograms. (A) macro-RAFT, (B) PVK, (C) PAAMP-*b*-PVK (A), (D) PAAMP-*b*-PVK (B), (E) PAAMP-*b*-PVK (C), (F) BL PAni.

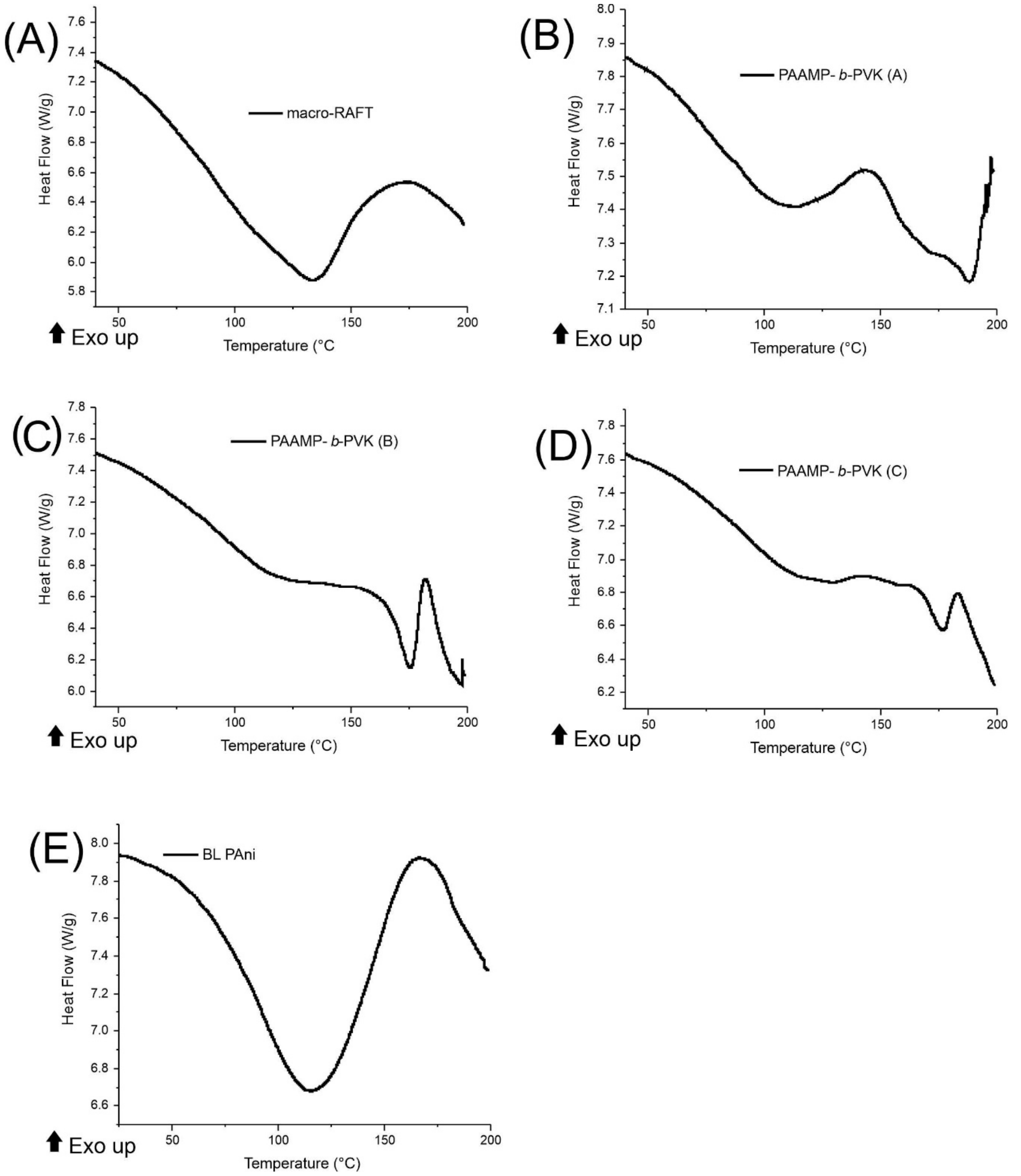

Figure S10. DSC thermograms of (A) macro-RAFT, (B) PAAMP-*b*-PVK (A), (C) PAAMP-*b*-PVK(B), (D) PAAMP-*b*-PVK (C), (E) BL PAni.
